# Avoidable waste of research related to outcome planning and reporting in clinical trials

**DOI:** 10.1186/s12916-018-1083-x

**Published:** 2018-06-11

**Authors:** Youri Yordanov, Agnes Dechartres, Ignacio Atal, Viet-Thi Tran, Isabelle Boutron, Perrine Crequit, Philippe Ravaud

**Affiliations:** 1 INSERM, U1153, Hôpital Hôtel-Dieus, 1, place du parvis Notre Dame, 75004 Paris, France; 2 Sorbonne Universités, UPMC Paris Univ-06, Paris, France; 3 Service des Urgences - Hôpital Saint Antoine, Assistance Publique–Hôpitaux de Paris (APHP), Paris, France; 4 Centre d’Épidémiologie Clinique, Hôpital Hôtel Dieu, Assistance Publique–Hôpitaux de Paris (APHP), Paris, France; 5 Faculté de Médecine, Université Paris Descartes, Sorbonne Paris Cité, Paris, France; 6 Cochrane France, Paris, France; 7 Department of Epidemiology, Columbia University, Mailman School of Public Health, New York, USA

**Keywords:** Randomized controlled trial, Waste of research, Outcome, Selective reporting, Core outcome set

## Abstract

**Background:**

Inadequate planning, selective reporting, and incomplete reporting of outcomes in randomized controlled trials (RCTs) contribute to the problem of waste of research. We aimed to describe such a waste and to examine to what extent this waste could be avoided.

**Methods:**

This research-on-research study was based on RCTs included in Cochrane reviews with a summary of findings (SoF) table. We considered the outcomes reported in the SoF tables as surrogates for important outcomes for patients and other decision makers. We used a three-step approach. (1) First, in each review, we identified, for each important outcome, RCTs that were excluded from the corresponding meta-analysis. (2) Then, for these RCTs, we systematically searched for registrations and protocols to distinguish between inadequate planning (an important outcome was not reported in registries or protocols), selective reporting (an important outcome was reported in registries or protocols but not in publications), and incomplete reporting (an important outcome was incompletely reported in publications). (3) Finally, we assessed, with the consensus of five experts, the feasibility and cost of measuring the important outcomes that were not planned. We considered inadequately planned or selectively or incompletely reported important outcomes as avoidable waste if the outcome could have been easily measured at no additional cost based on expert evaluation.

**Results:**

Of the 2711 RCTs included in the main comparison of 290 reviews, 2115 (78%) were excluded from at least one meta-analysis of important outcomes. Every trial contributed to 55%, on average, of the meta-analyses of important outcomes. Of the 310 RCTs published in 2010 or later, 156 were registered. Inadequate planning affected 79% of these RCTs, whereas incomplete and selective reporting affected 41% and 15%, respectively. For 63% of RCTs, we found at least one missing important outcome for which the waste was avoidable and for 30%, the waste was avoidable for all important outcomes.

**Conclusions:**

Most of the RCTs included in our sample did not contribute to all the important outcomes in meta-analyses, mostly because of inadequate planning or incomplete reporting. A large part of this waste of research seemed to be avoidable.

**Electronic supplementary material:**

The online version of this article (10.1186/s12916-018-1083-x) contains supplementary material, which is available to authorized users.

## Background

Clinical trials are only as credible as their outcomes, so to inform decision-making appropriately, randomized controlled trials (RCTs) must evaluate the outcomes that are most important to patients and their caregivers [1–8]. Failure to do so could contribute to the overwhelming problem of waste in research [9–16]. Waste of research related to inadequate outcome planning, selective reporting, and incomplete reporting of outcomes in RCTs prevents patients and their physicians from making well-informed decisions, with potential serious consequences if ineffective or harmful treatments are promoted [9, 12–14, 17, 18].

Therefore, when planning an RCT, researchers are expected to measure all important outcomes [9]. However, previous studies found that less than one-fifth of diabetes RCTs and less than one-quarter of cardiovascular trials considered patient-important outcomes as their primary outcomes [19, 20]. Instead, researchers frequently rely on surrogates as a proxy for final patient-important outcomes because these outcomes allow for smaller, faster, and thus, cheaper clinical trials [21–24]. However, surrogates can be misleading, because they may show exaggerated treatment effect sizes or even an apparent benefit of harmful treatments, as was the case for the use of antiarrhythmic drugs after myocardial infarction, which led to the deaths of several thousand patients decades ago [25–28].

Selective reporting has been repeatedly described as another important issue affecting RCT outcomes [29–32]. Outcome reporting bias arises when outcomes are selectively reported based on the nature and direction of the results [33]. In a recent study, the median proportion of RCTs with discrepancies between registered and published primary outcomes was 31% [34]. Statistically significant outcomes were 2 to 4 times more likely to be reported in publications than non-significant ones, which biases the available body of evidence toward more positive results [30].

Similarly, a median of 31% to 50% of efficacy outcomes were found to be incompletely reported in RCT articles [29, 35, 36]. With incomplete reporting of outcomes, outcomes cannot be included in meta-analyses, which poses a serious threat to the usability of trial results: not including all available results in meta-analyses can lead to a truncated vision of the overall body of evidence.

The purpose of this study was to describe the waste of research related to inadequate planning, selective reporting, or incomplete reporting of outcomes in RCTs and to examine to what extent this waste could be avoided. For this, we addressed the following specific questions: (1) What proportion of RCTs are excluded from meta-analyses due to outcome reasons? (2) Were the exclusions related to inadequate planning, selective reporting, or incomplete reporting? (3) Was it feasible to measure the missing outcomes at the planning stage, and at what cost?

## Methods

We performed a research-on-research study based on RCTs included in Cochrane systematic reviews. We used the outcomes reported in the summary of findings (SoF) tables of reviews as surrogates for important outcomes, because Cochrane systematic review SoF tables should include the most important outcomes for patients and other decision makers, whether they are available in RCTs or not [7]. This study used a three-step approach. (1) First, for all important outcomes, we identified the RCTs that were included in the Cochrane reviews but excluded from the corresponding meta-analyses because the important outcome was missing. (2) Then, we systematically searched for trial registrations and protocols to distinguish between the outcomes that were not planned (not included in registries or protocols, i.e., inadequate planning) and outcomes that were planned. For the planned outcomes, we distinguished between those that were adequately reported and those that were incompletely reported (poor reporting) or not reported (selective reporting). (3) Finally, we assessed, via expert consensus, the feasibility and cost of measuring the outcomes that were not planned.

The eligibility criteria at each phase of the study are summarized in Additional file 1.

### Identification of RCTs excluded from meta-analyses

#### Data sources

We obtained data from all Cochrane systematic reviews published between March 2011 and September 2014. They were provided by the Cochrane Collaboration editorial unit as XML files and contained all information reported by the review authors in RevMan, the software developed by the Cochrane Collaboration for preparing and maintaining systematic reviews [37]. Cochrane systematic reviews of interventions are organized by comparisons of two treatment groups. Meta-analyses are then performed for one or more outcomes within each comparison. Cochrane reviewers are encouraged to present an SoF table summarizing information on the quality of evidence and treatment effect magnitude (from the meta-analysis result) for the most important outcomes (see example in the review by Mocellin and colleagues [38]) [7]. According to the Cochrane collaboration, these outcomes should be important to all research end users, that is, patients and other decision makers [7]. These outcomes include a “wide variety of events such as mortality and major morbidity (such as stroke and myocardial infarction); however, they may also represent frequent minor and rare major side effects, symptoms and quality of life, burdens associated with treatment, and resource issues (costs)” [7].

#### Selection of relevant Cochrane systematic reviews

Using R 3.1.1 and the XML package, one of us (IA) removed from the set of reviews provided by the Cochrane Collaboration withdrawn Cochrane reviews and then identified all reviews of RCTs with an SoF table. We excluded reviews that included observational studies and those including only RCTs published before 2007 because we focused on recent RCTs. From all eligible Cochrane reviews, using a random number generator, we drew a random sample of 300 for an in-depth evaluation but excluded a further 10 reviews because their SoF tables mixed various interventions, which resulted in a final sample of 290 Cochrane reviews.

#### Identification of important outcomes

We considered outcomes reported in the SoF table as a surrogate for important outcomes. Therefore, we manually identified and extracted all outcomes reported in the SoF tables. Most reviews had a single SoF table, but some had several tables, corresponding to different comparisons. In this case, we focused on the main comparison as acknowledged by the authors. If the main comparison was not reported by the review authors or if various comparisons were reported in the same SoF table, we selected the comparison with the most outcomes and included the largest number of RCTs. If the SoF table reported various interventions (e.g., presented three meta-analyses of different outcomes for three different interventions), the review was excluded.

We classified each outcome as follows: mortality, other clinical event (e.g., myocardial infarction or stroke), therapeutic decision (e.g., transfusion), function (e.g., disability), pain, quality of life, adverse events or side effects (identified as such by the review authors), physiological variable (e.g., blood pressure or weight), biological variable (e.g., cholesterol levels), radiological variable (e.g., measure of joint space), compliance (e.g., discontinuation for any reason), process (e.g., duration of surgical procedure), resource use (hospitalization), cost-effectiveness, and satisfaction with care [39].

#### Identification of RCTs excluded from meta-analyses

For each review and for each important outcome, we identified all RCTs excluded from the outcome in the corresponding meta-analysis. To do so, by using the reference list of all studies included in the Cochrane review, we first identified all RCTs available for the selected comparison by extracting all RCTs included in any meta-analysis reported for this comparison. Then, for each of these RCTs, we manually evaluated whether they contributed to the meta-analysis of each important outcome by screening RCTs included in the corresponding meta-analysis.

### Evaluation of the reason for a missing outcome

We a priori hypothesized that the exclusion of an RCT from a meta-analysis for outcome reasons could be related to inadequate planning (i.e., the outcome was not planned to be measured), selective reporting (i.e., the outcome was planned but not reported in the publication), or incomplete reporting (i.e., the outcome was reported in the publication but not in a way that allowed for pooling of data).

To distinguish among inadequate planning, selective reporting, or incomplete reporting, for each RCT excluded from at least one meta-analysis, we screened the data available in Cochrane reviews and systematically searched for trial registration and/or protocols.

We focused on RCTs published in 2010 or later to maximize the chance of identifying trial registration or protocols.

#### Search for RCT registration and protocols

For each RCT, we individually assessed all available reports and information. To do that, we extracted the references identified from the “References to studies included in this review” section of the Cochrane reviews. One of us (YY) retrieved all the articles, conference abstracts, reports etc. related to the identified RCT. We screened all articles for any information regarding registration (name of trial registry and/or registration number) and/or protocol availability. When no reference to a trial registration was found, we used the following approach:

1. We searched the full text of the Cochrane review for any information regarding trial registration.
2. If no information was found, we searched the World Health Organization (WHO) International Clinical Trials Registry Platform (ICTRP). Also, according to the author’s affiliation, we searched the local registry if not part of the WHO ICTRP. We used the article title, the first and last author’s name as author or as investigator, or the author’s affiliation as search keywords.
3. If no information was found, we searched Google with the publication title and keywords regarding registration (e.g., registry, registration, NCT etc.).
4. If no information was found, we contacted the corresponding author to ask whether the trial was registered and whether a protocol was available.

#### Evaluation of the reason for the missing outcome

For each identified RCT, one of us (YY) classified each missing outcome into one of the following five categories:

1. Inadequate planning
  The outcome was not planned according to the protocol or the registry entry. It was not reported in the available trial reports.
2. Selective reporting
  The outcome was planned according to the protocol or the registry entry but was not reported in the available trial reports.
3. Incomplete reporting
  The outcome was planned according to the protocol or the registry entry and was reported in the available trial reports but incompletely or not in a way that allowed for meta-analysis (e.g., mixed model analyses reported, no results per group, missing control group results, difference in means without the standard error, etc.).
  No protocol or registry entry was found. The outcome was reported in the available trial reports but not in a way that allowed for meta-analysis.
4. Unable to distinguish between selective reporting and inadequate planning
  No protocol or registry entry was found and the outcome was not reported in the available trial reports.
5. Other situations
  The outcome was listed in the trial reports, but there was no event (e.g., the outcome was death but no death occurred).
  The outcome concerned adverse events, but there was no event (e.g., the outcome was “Major Complications—Visceral injury,” but no complications were reported).
  The outcome was reported in the available trial reports in a way that could allow for inclusion in a meta-analysis but was not included in the meta-analysis.

As a quality assessment measure, 10% of the RCTs were classified independently by two reviewers (YY and AD), with no disagreements.

### Evaluation of research waste

#### Feasibility and cost of measuring the missing outcomes that were not planned

To evaluate whether the missing outcomes that were not planned could have been easily measured, we used an expert consensus approach. One of us (YY) presented to an expert panel of five methodologists and trialists (AD, IB, PC, PR, and VT) each RCT for which at least one missing outcome was classified as not planned. After evaluating standardized information on the population, interventions in the experimental and control group, and other outcomes evaluated, the panel of experts were asked to answer the following questions regarding the missing outcomes:

- “According to you, given the other outcomes measured in the trial, and based on your experience, would you consider that measuring the presented outcome would be easy, moderately easy, difficult or impossible in most cases from a trialist’s perspective?”
- “According to you, given the other outcomes measured in the trial, and based on your experience, would you consider that measuring the presented outcome would be easy, moderately easy, difficult or impossible in most cases from a patient’s perspective?”
- “According to you, given the other outcomes measured in the trial, and based on your experience, what would be the approximate cost of measuring this outcome: no cost defined as ≤ 1% of the total cost of the trial; minor cost, defined as ≤ 5%; moderate cost, 5% to 15%; or major cost, 15% or more”. These percentages were indicative.
- “According to you, how important is the missing outcome: major importance; non-major importance?”

One of us (YY) ensured that all experts first gave their opinion and then discussed together, to avoid the opinion of one leading person influencing the others. The final feasibility and cost evaluation of every proposed adjustment was based on the group consensus.

The qualifications and areas of expertise of the experts involved are summarized in Additional file 2.

#### Avoidable waste of research related to missing outcomes

We defined avoidable waste related to missing outcomes as missing important outcomes related to:

- Selective reporting
- Incomplete reporting
- Inadequate planning, if the outcome was judged by the expert panel as easy to collect from both the trialist and patient perspective, not costly (i.e., no or minor additional cost), and of critical importance

At the trial level, such waste could have been partially avoided if the trial could have been included in the meta-analysis of at least one missing outcome and totally if it could have been included in all meta-analyses of missing outcomes.

### Statistical analysis

The analysis was mainly descriptive. Continuous data are presented as median (Q1–Q3) and categorical data as frequencies (%, 95% confidence interval [CI]). All analyses involved use of R 3.0.2 (2013–09-25) (R Foundation for Statistical Computing, Vienna, Austria. http://www.r-project.org/).

## Results

### Identification of RCTs excluded from meta-analyses

#### Selection and characteristics of the Cochrane systematic reviews

**Fig. 1:**
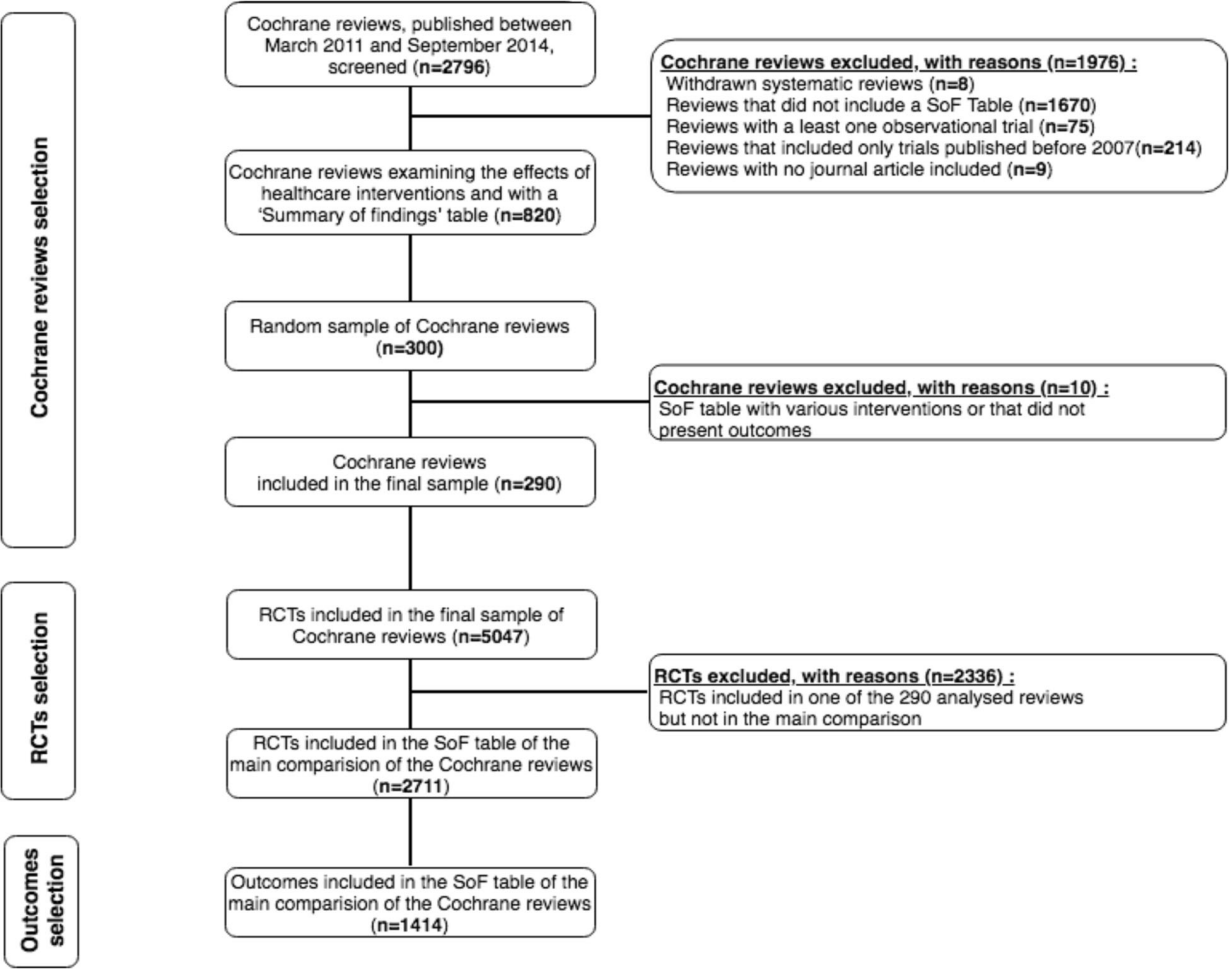
Flow chart of the selection process. This figure summarizes the selection process for Cochrane reviews, RCTs, and outcomes. The analyzed sample involved 290 Cochrane reviews, which included 2711 RCTs in the SoF table of the main comparison. The SoF tables reported 1414 important outcomes. RCT randomized controlled trial, SoF summary of findings

The complete selection process is described in Fig. 1. Briefly, from the 2796 Cochrane systematic reviews published between March 2011 and September 2014, 820 reviews corresponded to our eligibility criteria. Our selection process resulted in a sample of 290 reviews (5047 RCTs), with a median of 11 RCTs per review (Q1–Q3: 5–21) (Fig. 1). The subset of included reviews appeared comparable to the eligible Cochrane Reviews (Additional file 3). The reviews investigated 47 different health research topics, the most common being airways (7%, *n* = 21/290), menstrual disorders and subfertility (7%, *n* = 20/290), and anesthesia (6%, *n* = 18/290) (Table 1). Experimental interventions were pharmacological in 60% of reviews (*n* = 175/290).

**Table 1:** Characteristics of selected systematic reviews and outcomes

Review characteristics	*n* = 290
	Median (Q1–Q3)
No. of trials per review	11 (5–21)
No. of trials in the main comparison	5 (3–12)
No. of comparisons per review	2 (1–5)
Cochrane review groups	*n* = 290
	No. (%)
Airways	21 (7)
Menstrual disorders and subfertility	20 (7)
Anesthesia	18 (6)
Oral health	15 (5)
Schizophrenia	15 (5)
Hepato-biliary	12 (4)
Neuromuscular disease	12 (4)
Musculoskeletal	11 (4)
Infectious diseases	10 (3)
Other	156 (54)
Type of assessed intervention	*n* = 290
	No. (%)
Pharmacological	175 (60)
Non-pharmacological	115 (40)
Outcomes characteristics	*n* = 141
No. of outcomes per SoF table, median (Q1–Q3)	5 (3–7)
No. of trials per meta-analysis, median (Q1–Q3)	3 (2–7)
Outcome categories	*n* = 1414
	No. (%)^*^
Function	384 (27)
Other clinical events	198 (14)
Adverse events; side effects	174 (12)
Mortality	138 (10)
Quality of life	98 (7)
Biological variables	89 (6)
Process, resource use	74 (5)
Pain	71 (5)
Physiological variables	56 (4)
Compliance	45 (3)
Therapeutic decision	33 (2)
Satisfaction with care	24 (2)
Radiological variables	23 (2)
Cost-effectiveness	16 (1)

*The total exceeds 100% because some outcomes were included in more than one category

The reviews included a median of 5 outcomes per SoF table for the main comparison (Q1–Q3: 3–7, Min–Max: 1–12), for a total of 1414 outcomes. Mortality represented 10% (*n* = 138/1414) of the outcomes, and other clinical events, 14% (*n* = 198/1414); quality of life, 7% (*n* = 98/1414); function, 27% (*n* = 384/1414); and adverse events, 12% (*n* = 174/1414) (Table 1). Biological variables, process and resource use, and physiological variables accounted for 6% (*n* = 89/1414), 5% (*n* = 74/1414), and 4% (*n* = 56/1414), respectively. The corresponding meta-analyses included a median of 3 RCTs (Q1–Q3: 2–7), maximum 76.

#### Proportion of RCTs excluded from the meta-analyses due to outcome reasons

The 290 reviews included a total of 2711 RCTs in the main comparison; 596 (22%; 95% CI: 20–24) were included in all meta-analyses of important outcomes, and 2115 (78%, 95% CI: 76–80) were excluded from at least one meta-analysis. Every RCT contributed to 55%, on average, of the meta-analyses for important outcomes.

### Evaluation of the reason for missing outcomes

Among the 2115 RCTs excluded from at least one meta-analysis of important outcomes, 310 were published in 2010 or later. We further excluded 19 RCTs, because their reports were not accessible (e.g., retracted paper) or were not in English or French (e.g., Chinese), which left 291 RCTs for further evaluation, with a total of 971 missing outcomes. A registration number or a protocol was retrieved for 54% (*n* = 156/291) of these RCTs (corresponding to 461 missing outcomes).

Reasons for missing outcomes were incomplete reporting for 21% of missing outcomes (*n* = 204/971) in 40% of RCTs (*n* = 117/291), and inadequate planning for 29% of missing outcomes (*n* = 282/971) in 42% of RCTs (*n* = 123/291) (Table 2). Confirmed selective reporting represented 4% of missing outcomes (*n* = 36/971) in 9% of RCTs (*n* = 25/291). Nevertheless, 42% of RCTs (*n* = 122/291) had no protocol or registration, so for these, we could not distinguish between selective reporting and inadequate planning.

**Table 2:** Classification of reasons for missing outcomes

	All trials published in 2010 or later			
Reasons for missing outcome	All trials (registered and unregistered)		Registered trials	
	No. of affected outcomes (%) *N* = 971	No. of affected trials (%)^*^ *N* = 291	No. of affected outcomes (%) *N* = 461	No. of affected trials (%)^*^ N = 156
Inadequate planning	282 (29)	123 (42)	282 (61)	123 (79)
Selective reporting	36 (4)	25 (9)	34 (7)	23 (15)
Incomplete reporting	204 (21)	117 (40)	98 (21)	64 (41)
Unable to distinguish between selective reporting and lack of planning	363 (39)	122 (42)		
Other situations	86 (9)	63 (24)	47 (10)	41 (26)

*The total exceeds 100% because some outcomes were included in more than one category

When restricting our description to registered RCTs (*n* = 156), inadequate planning concerned 61% of missing outcomes (*n* = 282/461) in 79% of RCTs (*n* = 123/156), whereas incomplete reporting accounted for 21% of missing outcomes (*n* = 98/461) in 41% of RCTs (*n* = 64/156), and selective reporting, 7% of missing outcomes (*n* = 34/461) in 15% of RCTs (*n* = 23/156) (Table 2).

### Evaluation of research waste

#### Feasibility of measuring the missing outcomes that were not planned

We submitted the 282 outcomes missing due to inadequate planning from 123 RCTs to our panel of experts. For 78% of outcomes (*n* = 221/282), the experts judged the outcome of critical importance given the context. For these 221 critically important outcomes, they considered that 82% (*n* = 182) could have been easily measured from both the trialist and patient perspective at no cost (Additional file 4).

#### Avoidable waste of research due to missing important outcomes

**Fig. 2:**
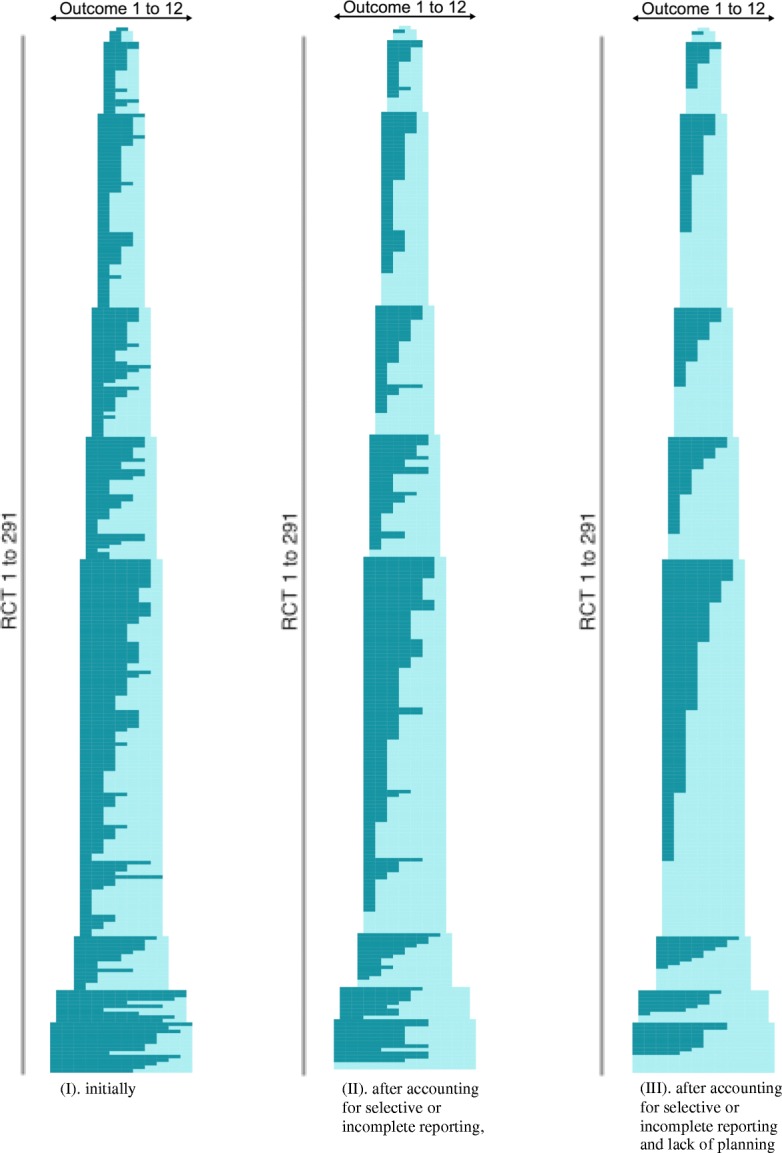
Avoidable waste of research related to missing outcomes in RCTs published in 2010 or later and not contributing to all meta-analyses of important outcomes (*n* = 291). This figure summarizes the evolution of the outcomes status of SoF tables (O1 to O12 because SoF tables included from 1 to 12 outcomes per review) for each RCT at the different steps of our study: step I (i.e., information as it was extracted), step II (i.e., after evaluating the reason for the missing outcome) and after considering if there was no selective reporting or incomplete reporting, and finally step III (i.e., evaluation of waste of research) and after accounting for inadequate planning and selective reporting or incomplete reporting (i.e., adequate planning and no selective reporting or incomplete reporting). On the *y*-axis, each line represents one of the 291 RCTs published in 2010 or later and not contributing to all meta-analyses of the outcomes reported in the SoF table. The *x*-axis represents the outcomes reported in the SoF table. Each brick represents a single outcome of the SoF table. The color is a visual representation of the presence or absence of the outcome in the RCT. Light blue means that the outcome was present in the corresponding RCT and dark blue that the outcome was absent in the RCT. RCT randomized controlled trial, SoF summary of findings

For the 291 RCTs published in 2010 or later, taking into account selective or incomplete outcome reporting, waste of research could have been partially avoided for 43% (*n* = 126) and totally (i.e., the trial could have been included in all meta-analyses of important outcomes) for 12% (*n* = 34) (Fig. 2). If we also consider missing outcomes that could have been easily measured from the planning stage at no additional cost as judged by our experts, waste of research could have been partially avoided for 63% of RCTs (*n* = 183) and totally for 30% (*n* = 86) (Fig. 2).

## Discussion

We evaluated the avoidable waste of research due to outcome-related reasons across a large number of RCTs included in recent Cochrane systematic reviews in a variety of medical fields. Our analysis revealed that 78% of the RCTs were not included in all meta-analyses of important outcomes and that every RCT contributed to 55%, on average, of the meta-analyses of these important outcomes. Among registered RCTs, inadequate planning was the most common reason affecting 79% of RCTs with missing outcomes. Such waste could have been partially avoided for 63% of the RCTs and totally for 30%.

Our findings suggest that many RCTs were not included in all meta-analyses of important outcomes and that every RCT contributed to only half of the meta-analyses, on average. These results seem consistent with the literature. In a recent study of patient-important outcomes, the median proportion of trials included in a meta-analysis for such outcomes was about 60% [39]. Approximately half of the RCTs included in another sample of Cochrane reviews were included in the pooled effect size estimates in the meta-analyses of patient-important outcomes [40], whereas more than one-third of the outcomes pre-specified in the review were not reported in the results sections [18, 41].

Although possible reasons for excluding RCTS from meta-analyses have been hypothesized, the respective contribution of inadequate planning and selective reporting or incomplete reporting was unknown. Our results provide major insights into this question, showing that many RCTs do not plan the most important outcomes to evaluate. Inadequate planning represents a missed opportunity within a trial because equipoise remains unchanged after this trial. However, a missed opportunity at the trial level will result in waste of research at the meta-analysis level. Actually, clinical research should be considered a sequential process, with each new trial contributing to the existing body of evidence. If a trial fails to do so because of a missed opportunity, it becomes a source of waste of research and resources at the meta-analysis level because this trial will not contribute to the overall evidence. Cochrane reviewers may select outcomes that differ in importance from those selected by trialists (typically those defined as primary outcomes and used for sample size calculation). Trialists rarely consider adverse events (particularly severe adverse events) as primary outcomes and for sample size calculation. However, these outcomes are crucial to assess and report because they could be included in meta-analyses to increase power and inform decision-making. Identifying important outcomes is challenging, and without a consensus on outcomes, heterogeneity in outcomes evaluated will remain [41].

To overcome these issues, several initiatives have emerged to improve the relevance and consistency of outcomes used in clinical research [4, 42–45]; one is the Core Outcome Measures in Effectiveness Trials (COMET) initiative promoting the development and use of standardized core outcome sets (COSs). COMET offers extremely helpful tools for researchers aiming to develop COSs in their fields. However, although the number of COSs is rapidly increasing, it remains limited [46–48], and searching for specific existing COSs for a given condition is difficult. The COMET website may evolve to present available COSs in a tabular fashion to help trialists identify the outcomes to use and researchers the conditions for which COSs are needed. Another perspective would be to use the outcomes reported in the SoF table of Cochrane reviews to develop COSs, because most of these outcomes seem important to patients.

Incomplete reporting was another common reason for outcomes excluded from meta-analyses. Two main situations can be distinguished. First, the failure to report results adequately (e.g., reporting means without standard deviations) and second, the reporting of an analysis that cannot be included as such in a meta-analysis (e.g., reporting of repeated data analysis). The first situation is clearly related to poor reporting. We previously showed that results were more completely reported at ClinicalTrials.gov than in publications, probably because of the standardized template used to report results at ClinicalTrials.gov [49]. Such standardized templates may help trialists determine which data should be reported and should be included in reporting guidelines and required by journals to improve the presentation of results. The other situation is more complex because it does not reflect poor reporting, just reporting in a different way. Studies with repeated data are meant to record measurements at numerous time points to inform researchers of changes over time. However, several approaches for meta-analyzing these types of data exist, and they can differ in terms of the data needed for analysis [50]. Therefore, reporting trial results not just for an immediate use but also considering a later inclusion in meta-analyses can be challenging. The Instrument for reporting of Planned Endpoints in Clinical Trials (InsPECT) reporting guidelines (currently under development, https://www.inspect-statement.org/) might help trial authors mitigate the effects of incomplete reporting in RCTs.

Finally, selective reporting of outcomes seems to affect a few trials. It remains an important issue in clinical trials, with, on average, one-third of discrepancies in primary outcomes between protocols and publications [29] or between registry information and publications [31]. In our situation, this concerns only outcomes that were planned and not reported at all in publications. Actually, reviewers consider a given outcome whether this outcome is reported as a primary or a secondary outcome.

### Strengths and limitations of the study

In this study, we used an original approach to evaluate the main reasons why a trial was not included in a meta-analysis and the part of this waste that could be avoided. We analyzed a large set of trials included in a vast, unselected, sample of recent Cochrane reviews exploring various health-care research topics. Assembling numerous experts in such a variety of fields to identify the most important outcomes to evaluate would have been challenging. As a proxy, we used the outcomes reported in the SoF tables that were considered by the review authors as the most important to measure for a given comparison in a particular health condition [7]. We used the consensus of an expert panel of recognized methodologists and trialists to assess the feasibility and costs of measuring the missing outcomes. To avoid overestimating waste of research, we also asked the panel to evaluate the importance of outcomes and considered that research was wasted for only those outcomes the panel confirmed to be of critical importance.

Our study has several limitations. First, we focused only on trials included in Cochrane reviews published between 2010 and 2014. However, it has been reported that Cochrane and non-Cochrane reviews have systemic differences, likely reflective of different methodology [51]. Then, we focused on trials included in the reviews but excluded from at least one meta-analysis. We did not consider the number of trials excluded from the reviews for outcome-related reasons. Therefore, we probably underestimated the proportion of trials excluded from meta-analyses for outcome-related reasons. Despite the recommendation of the Cochrane Handbook to determine trial eligibility independently of outcomes measured (studies should not be excluded just because they provide no usable data), some trials may be excluded for this reason [7]. Thus, although, our sample of RCTs was from a large variety of medical fields, the database used (Cochrane reviews published between 2010 and 2014 and with an SoF table), the limited sample size, the language restrictions, or the expertise of our panel of experts may limit the generalizability of our results. Finally, as a quality measure, 10% of the RCTs were classified independently by two reviewers, since this assessment is challenging and implies some subjectivity.

## Conclusions

Our study shows that most RCTs included in our sample of Cochrane reviews did not contribute to all meta-analyses of the most important outcomes mainly because of inadequate planning or incomplete reporting. Such waste could have been partially avoided for 63% of the trials and totally for 30%. We need to accelerate the development and dissemination of COSs and reduce poor outcome reporting to avoid this waste.

## Additional files

Additional file 1: Summary of the inclusion and exclusion criteria for the different phases of the study. (DOCX 18 kb)

Additional file 2: Qualifications and areas of expertise of the experts involved in the final step of the study. (DOCX 15 kb)

Additional file 3: Characteristics of the 820 Cochrane systematic reviews and the analyzed subset of 290 reviews. (DOCX 15 kb)

Additional file 4: Experts’ opinion of the feasibility and costs of measuring the missing outcomes. (DOC 56 kb)

## Abbreviations

CI: Confidence interval
COMET: Core Outcome Measures in Effectiveness Trials
COS: Core outcome sets
ICTRP: International Clinical Trials Registry Platform
RCT: Randomized controlled trial
SoF: Summary of findings
WHO: World Health Organization
