# Supplementary material for: Avoidable waste of research related to outcome planning and reporting in clinical trials
Source: BMC Med. 2018 Jun 11;16:87. doi: 10.1186/s12916-018-1083-x (PMC5994653; doi:10.1186/s12916-018-1083-x)
Supplement: Supplementary file 1 — Summary of the inclusion and exclusion criteria for the different phases of the study. (DOCX 18 kb) [file 12916_2018_1083_MOESM1_ESM.docx]

**Additional file 1:** Summary of the inclusion and **exclusion** criteria at the different phases of the study

| **Methods phase** | **Inclusion criteria** | **Exclusion criteria** |
| --- | --- | --- |
| **I. Identification of RCTs excluded from meta-analyses** | Cochrane reviews:   - Examining the effects of healthcare interventions (pharmaceutical and non-pharmaceutical) - Published between March 2011 and September 2014 - Reporting a Summary of findings table (SoF).   RCTs:   - Included in at least one meta-analysis of the main comparison - Excluded from at least one meta-analysis of an important outcome (i.e., an outcome reported in the SoF table) | Cochrane reviews:   - Withdrawn - Including observational studies - Including only trials published before 2007   RCTs:   - Not included in any meta-analyses of the main comparison |
| **II. Evaluation of the reason for missing outcome** | RCTs   - Published in 2010 or later | RCTs   - With inaccessible reports (e.g. retracted publications) - Published in languages other than English or French. |
| **III. Evaluation of research waste** | RCTs   - With at least one outcome classified as “Selective reporting” at phase II. - With at least one outcome classified as “Incomplete reporting” at phase II. - With at least one outcome classified as “Inadequate planning” at phase II. |  |
