# Supplementary material for: Avoidable waste of research related to outcome planning and reporting in clinical trials
Source: BMC Med. 2018 Jun 11;16:87. doi: 10.1186/s12916-018-1083-x (PMC5994653; doi:10.1186/s12916-018-1083-x)
Supplement: Supplementary file 2 — Qualifications and areas of expertise of the experts involved in the final step of the study. (DOCX 15 kb) [file 12916_2018_1083_MOESM2_ESM.docx]

**Additional file 2:** Qualifications and areas of expertise of the experts involved in the final step of the study

The 5 experts involved in the final step of our study were all physicians and researchers, with different medical specialties and various backgrounds.

1. Dr Agnes Dechartres is a public health specialist, has published many meta-epidemiological or methodological studies, and has been involved as a methodologist in several clinical trials.
2. Pr Isabelle Boutron is a rheumatologist, has published many studies pertaining to research on research and has been involved as a methodologist in several clinical trials.
3. Dr Perrine Crequit is a pneumologist and has published several studies in the field of pneumology, oncology and systematic reviews. She participated as a local investigator of several clinical trials.
4. Pr Philippe Ravaud is a rheumatologist and has published many clinical trials and studies pertaining to research on research. He has been involved as a PI and a methodologist in numerous clinical trials.
5. Dr Viet-Thi Tran is a general practitioner and has worked extensively on topics such as the burden of treatment or treatment adherence in multimorbid patients. He was involved in several clinical trials, including studies led in challenging areas such as Sub-Saharan Africa.
