# Supplementary material for: Avoidable waste of research related to outcome planning and reporting in clinical trials
Source: BMC Med. 2018 Jun 11;16:87. doi: 10.1186/s12916-018-1083-x (PMC5994653; doi:10.1186/s12916-018-1083-x)
Supplement: Supplementary file 3 — Characteristics of the 820 Cochrane systematic reviews and the analyzed subset of 290 reviews. (DOCX 15 kb) [file 12916_2018_1083_MOESM3_ESM.docx]

**Additional file 3:** Characteristics of the 820 Cochrane systematic reviews and the analysed subset of 290 reviews:

|  | **All eligible reviews** | **Sample** |
| --- | --- | --- |
| **Cochrane review groups** | **N=820** | **N=290** |
|  | **No. (%)** | **No. (%)** |
| Airways | 67 (8) | 21 (7) |
| Menstrual disorders and subfertility | 51 (7) | 20 (7) |
| Anaesthesia | 46 (6) | 18 (6) |
| Hepato-biliary | 43 (5) | 12 (4) |
| Oral health | 37 (5) | 15 (5) |
| Infectious diseases | 29 (4) | 10 (3) |
| Musculoskeletal | 28 (3) | 11 (4) |
| Schizophrenia | 28 (3) | 15 (5) |
| Endocrinology | 25 (3) | 7 (2) |
| Other | 466 (57) | 156 (54) |
