# Supplementary material for: Avoidable waste of research related to outcome planning and reporting in clinical trials
Source: BMC Med. 2018 Jun 11;16:87. doi: 10.1186/s12916-018-1083-x (PMC5994653; doi:10.1186/s12916-018-1083-x)
Supplement: Supplementary file 4 — Experts’ opinion of the feasibility and costs of measuring the missing outcomes. (DOC 56 kb) [file 12916_2018_1083_MOESM4_ESM.doc]

**Additional file 4:** Experts’ opinion of the feasibility and costs of measuring the missing outcomes

| **Measuring the outcome: trialist perspective** | **n=282** | |
| --- | --- | --- |
|  | **No. (%)** | |
| Easy | 227 (81) | |
| Moderately easy | 50 (17) | |
| Difficult | 3 (1) | |
| Impossible | 0 (0) | |
| Already available | 2 (1) | |
|  |  | |
| **Measuring the outcome: patient perspective** | **n=282** | |
|  | **No. (%)** | |
| Easy | 229 (82) | |
| Moderately easy | 48 (16) | |
| Difficult | 3 (1) | |
| Impossible | 0 (0) | |
| Already available | 2 (1) | |
|  |  | |
| **Measuring the outcome: cost evaluation** | **n=282** | |
|  | **No. (%)** | |
| No cost | 221 (79) | |
| Minor costs | 48 (16) | |
| Moderate costs | 11 (4) | |
| Major costs | 2 (1) | |
|  |  | |
| **Outcome importance** | **n=282** | |
|  | **No. (%)** | |
| Important | 221 (78) | |
| Not Important | 59 (21) | |
| Already available | 2 (1) | |
|  |  | |
| **How easy would it be to measure it as compared with the other measured or planned outcomes?** | **n=282** | |
|  | **No. (%)** | |
| Easier | 62 (22) | |
| As easy | 165 (59) | |
| More difficult | 53 (18) | |
| Already available | 2 (1) | |
|  |  | |
|  | |  |
